# Cross-over comparison and new chemotherapy regimens in metastatic pancreatic cancer

**DOI:** 10.1007/s12254-017-0352-2

**Published:** 2017-09-07

**Authors:** Markus Kieler, Matthias Unseld, Daniela Bianconi, Gerald W. Prager

**Affiliations:** Department of Medicine I, Division of Oncology, Comprehensive Cancer Center, Medical University Vienna – General Hospital, Waehringer Guertel 18–20, 1090 Vienna, Austria

**Keywords:** Pancreatic ductal adenocarcinoma, Chemotherapy, FOLFIRINOX, Gemcitabine and nab-paclitaxel, Nanoliposomal irinotecan

## Abstract

Despite decades of research, pancreatic ductal adenocarcinoma (PDAC) is still one of the most lethal malignant diseases with a devastating 5‑year overall survival of only 4–5%. Indeed, long-term survival was not affected by the introduction of new systemic cytotoxic chemotherapies which remain the key cornerstone in the treatment of metastatic PDAC. In the first-line setting, FOLFIRINOX based upon the results of the PRODIGE/ACCORD trial and gemcitabine with albumin-bound paclitaxel (GNP) based upon the MPACT trial have both been approved as therapeutic options for patients with no significant comorbidities and good performance status. As there is no direct comparison between these regimens, the choice in first-line treatment depends on the toxicity profile, patient’s preferences and reimbursability. In the second-line setting, the results of the NAPOLI-1 trial have led to the approval of nanoliposomal irinotecan (nal-iri) in combination with 5‑fluorouracil (5-FU) for the treatment of patients with mPDAC progressing under gemcitabine-based chemotherapy and therefore this regimen is the first to be approved for use in second-line therapy.

## Introduction

Despite decades of research, pancreatic ductal adenocarcinoma (PDAC) is still one of the most lethal malignant diseases with a devastating 5‑year overall survival of only 4–5%. Indeed, long-term survival was not affected by the introduction of new chemotherapies. PDAC is projected to become the second leading cause of cancer-related deaths before 2030 [1]. In comparison to other malignant diseases such as melanoma or non-small cell lung cancer that have witnessed the implementation of targeted therapies or immune-related drugs in daily clinical practice [2, 3], systemic cytotoxic chemotherapy remains the key cornerstone in the treatment of metastatic pancreatic cancer (mPDAC).

In 1997, gemcitabine became the standard treatment option when Burris and colleagues demonstrated a modest survival improvement and an increase in disease control rate from 4.2 to 23.8% compared with 5‑fluorouracil therapy [4]. After ten years of no significant survival benefit shown in any of the many clinical trials, Sultana and colleagues published two meta-analysis in 2007 and 2008, demonstrating a survival benefit for gemcitabine combinational therapies versus gemcitabine alone [5, 6]. Then with the increase in the understanding of PDAC genome, distinct alterations in certain signaling pathways were revealed [7]. The hope for broadly applicable molecular targeted therapies was not fulfilled because most of the genomic alterations like TP53, SMAD-4 and the most common KRAS-mutation is currently not targetable. With just one targeted agent, namely erlotinib that has demonstrated a small, but significant survival benefit in combination with gemcitabine compared to gemcitabine monotherapy, the golden age has not yet come for precision medicine in this disease [8].

## First-line chemotherapy in metastatic pancreatic cancer

In 2011, the results of the PRODIGE/ACCORD trial revealed that FOLFIRINOX (oxaliplatin, irinotecan, fluorouracil, and leucovorin) significantly increases the survival and quality of life at the cost of higher toxicity compared to monotherapy with gemcitabine in first-line treatment [9]. Two years later another multicenter randomized phase III trial (MPACT trial) was published and showed superior survival rates for the combinational treatment with gemcitabine and NAB(albumin bound)-paclitaxel (GNP) compared to monotherapy with gemcitabine [10]. These two new therapeutic options have been integrated in clinical practice guidelines such as from the European Society of Medical Oncology (ESMO), the American Society of Clinical Oncology (ASCO) and the National Comprehensive Cancer Network (NCCN) and are recommended both as regimens in the first-line treatment [11–13]. While in patients with significant comorbidities and an Eastern Cooperative Oncology Group (ECOG) performance status of 2 the optimal first-line treatment still remains monotherapy with gemcitabine, the best choice for first-line treatment in fit patients with an ECOG performance status of 0–1 has not yet been fully elucidated.

## FOLFIRINOX and gemcitabine with albumin-bound paclitaxel as first-line regimens in the treatment of mPDAC

Table 1 shows a listing of the core data of the two pivotal trials. In the PRODIGE/ACCORD trial, median overall survival (OS) with the experimental arm was 11.1 months compared with 6.8 months with gemcitabine (hazard ratio [HR], 0.57; 95% confidence interval (CI), 0.45 to 0.73; *p* = 0.001). In the MPACT, median OS with the experimental arm was 8.5 months compared with 6.7 months with gemcitabine (HR, 0.72; 95% CI, 0.62 to 0.83; *p* = 0.001).

**Table 1:** Tabular comparison of the FOLFIRINOX and NAB-paclitaxel trial

		FOLFRIRINOX vs. gemcitabine[9] (PRODIGE/ACCORD trial)	Gemcitabine/NAB-paclitaxel vs. gemcitabine[10] (MPACT trial)
Study characteristics	Study phase	II/III	III
	No. of patients	342	861
	Location	France (48 centers)	Multinational (151 centers)
Patient characteristics	Median age	61	62
	Female/male	38%/62%	43%/57%
	Performance status	ECOG 0 (37.4%)	KPS 100 (16%)
		ECOG 1 (61.9%)	KPS 80–90 (77%)
		ECOG 2 (0.6%)	KPS 60–70 (7%)
	Site of metastasis	Liver (87.6%)	Liver (85%)
		Lung (19.4%)	Lung (35%)
		Peritoneum (19.4%)	Peritoneum (4%)
Response and survival	OS	11.1 months	8.5 months
	PFS	6.4 months	5.5 months
	ORR	31.6%	23%
	DCR	70.2%	48%
	PR	31%	23%
	SD	38.6%	27%
Toxicity (Grade 3–4)	Treatment-related deaths	1%	4%
	Neutropenia	45.7%	38%
	Febrile neutropenia	5.4%	3%
	Thrombocytopenia	9.1%	13%
	Anemia	7.8%	13%
	Fatigue	23.6%	17%
	Peripheral neuropathy	9%	17%
	Diarrhea	12.7%	6%
	Alopecia	11.2%	50%
	Receipt of growth factors	42.5%	26%

*OS* overall survival, *PFS* progression free survival, *ORR* overall response rate, *DCR* disease control rate, *PR* partial response, *SD* stable disease

These trials are not comparable as they differ in certain points. The PRODIGE/ACCORD trial was designed as a clinical phase II trial which was consecutively extended to a clinical phase III trial conducted in 48 centers in France including 342 patients with mPDAC, whereas the GNP trial was designed as a multinational trial in 151 centers and enrolled 861 patients. Another point of criticism is that in the French trial no central radiological assessment has been performed. Two other facts that add to the controversy concerning the preferred treatment in the first-line setting is the age limit and better performance status of the patients in the PRODIGE/ACCORD trial. Patients older than 75 years and an ECOG performance status over 1 were excluded from the FOLFIRINOX trial. In contrast, the age of the patients in the GNP trial ranged from 27 to 86 years and nearly 42% of the patients presented with a Karnofsky performance status of under 90.

Quality of life (QoL) was measured in the PRODIGE/ACCORD trial and this regimen significantly reduces QoL impairment compared with gemcitabine [14], whereas QoL was not measured in the MPACT trial. However a Quality-adjusted Time Without Symptoms of disease progression or Toxicity methodology (Q-TWiST) analyses showed a significant gain in quality-adjusted survival by treatment with GNP compared with gemcitabine [15].

The safety profile of a chemotherapy is important when it comes to implementing it in the daily clinical practice. Concerning the hematological toxicity, the rates of grade 3 and 4 neutropenia and febrile neutropenia were higher in patients treated with FOLFIRINOX (45.7% and 5.4%) than in patients treated in the GNP arm (38% and 3%). This resulted in a much higher rate of G‑CSF(granulocyte-colony stimulating factor)-usage in the FOLFIRINOX arm (42.5%) compared to the rate in the GNP arm (26%). Grade 3/4 diarrhea was observed in 12.7% versus 6% of patients treated with FOLFIRINOX and GNP. For other side effects we refer to Table 1. Significant toxicity was observed in both trials, leading to the development of more tolerable regimens. Phase II trials with a modified FOLFIRINOX regimen for example leaving out the 5‑FU bolus and or a 25% of reduction of the 5‑FU bolus and irinotecan doses have demonstrated comparable efficacy like the regimen used in the phase III trial while at the same time reducing toxicity [16–18]. In an analysis of the GNP trial it was shown that patients with dose reductions and delays had better outcomes because these dose reductions were effective when necessary to ameliorate toxicity allowing greater treatment exposure without compromising efficacy [19].

Overall, both regimens have demonstrated a survival benefit and better quality of life compared with gemcitabine. As there is no direct comparison between these regimens, the choice in first-line treatment depends on the toxicity profile, age, patient’s preferences and reimbursability.

## New chemotherapies in second-line treatment of mPDAC

Before the implementation of more efficacious first-line treatment, there was little use for a second-line in mPDAC. For this reason the current level of evidence to support a particular regimen sequencing first-line treatment with GNP or FOLFRINOX is very low. There have been three phase III trials investigating 5‑FU in combination with oxaliplatin after failure of first-line treatment with gemcitabine. While the results of two trials (CONKO-01 and CONKO-003) suggested a survival benefit for treatment with 5‑FU and oxaliplatin [20, 21], in the PANCREOX trial survival of patients treated with either FOLFOX or 5‑FU alone did not show any differences [22]. The differences in the outcomes have to be interpreted in the context of the different chemotherapy regimens used, as patients in the CONKO trials were treated with OFF and in the PANCREOX trial with FOLFOX6. It is currently not clear if oxaliplatin has its role in second-line therapy and if it may still provide a valid option.

Favorable preclinical data on nanoliposomal irinotecan (nal-iri) and early phase trials led to the recently published phase III trial (NAPOLI-1) investigating liposomal irinotecan in patients with mPDAC progressing to at least to first-line treatment [23]. In all, 417 patients, who were previously treated with gemcitabine-based chemotherapy, were randomly assigned to receive either liposomal irinotecan monotherapy (120 mg/m^2^) every 3 weeks or 5‑FU (2000 mg/m^2^ over 24 h every week for the first 4 weeks of every 6‑week cycle). Median OS was 6.1 months in the combination arm and 4.2 months in patients assigned to the 5‑FU control arm (HR 0.67, 95% CI 0.49–0.92; *p* = 0.012). In patients who were allocated to liposomal irinotecan monotherapy, median OS was 4.9 months, which was not significantly different to the control arm (HR 0.99, 95% CI 0.77–1.28; *p* = 0.94). Based upon these results the United States Food and Drug Administration (FDA) and European Medicines Agency (EMA) approved nal-iri in combination with 5‑FU for the treatment of patients with mPDAC progressing under gemcitabine-based chemotherapy and therefore this regimen is the first to be approved for use in second-line therapy. However, it remains unclear how nal-iri should be used in patients that have received FOLFIRINOX in the first-line setting. Despite a clear preclinical reason for the use of nanoencapsulated drugs, nal-iri has not been compared with unencapsulated irinotecan in the treatment of mPDAC. Leaving these questions unanswered, the results from the NAPOLI-1 had an impact on the therapeutic landscape by offering a sequential therapy strategy for patients with mPDAC (Table 2).

**Table 2:** Summary of basic clinical data and results from the NAPOLI-1 trial

		Nal-iri plus 5‑FU	Nal-iri monotherapy	5-FU monotherapy
No. of patients	–	117	151	149
Median age, years	–	63	65	63
Female/male	–	41%/59%	42%/58%	46%/54%
Previous lines of chemotherapy	0	13%	11%	13%
	>1	53%	57%	58%
	≥2	34%	32%	30%
Karnofsky performance status	100	15%	15%	15%
	80–90	76%	75%	77%
	50–70	9%	10%	7%
Response and survival	OS	6.1 months	4.9 months	4.2 months
	PFS	3.1 months	2.7 months	1.5 months
	ORR	16%	6%	1%

*OS* overall survival, *PFS* progression free survival, *ORR* overall response rate

Based upon the available data and efficacious regimens, clinicians are now able to choose between two sequential treatment options. If FOLFIRINOX is used in first-line, gemcitabine with or without NAB-paclitaxel can be used in second-line treatment. However, there is limited data for administering GNP after failure of FOLFIRINOX. Two retrospective studies have evaluated this sequence and while the rate of grade 3 and 4 toxicities were quite high with up to 40% in one study, the reported median OS with 23 weeks and 8.8 months suggests that GNP may be an effective second-line regimen after failure of FOLFIRINOX [24, 25]. In the case of GNP as first-line treatment, patients could be offered an approved second-line treatment with nal-iri plus 5‑FU according to the NAPOLI-1 trial or with less evidence treatment with oxaliplatin plus 5‑FU (OFF regimen) according to the CONKO trials.

## Summary and perspective

The therapeutic management of patients with mPDAC has been changed by the results of the main trials discussed in this review. Systemic cytotoxic chemotherapy remains the cornerstone in the management of patients with mPDAC and clinicians now have two potential treatment sequences available for the first time in the management of this disease.
